# RstAB activates type 1 fimbriae to promote uropathogenic *Escherichia coli* bladder invasion

**DOI:** 10.1016/j.isci.2026.116333

**Published:** 2026-06-11

**Authors:** Qian Wang, Xinyu Gao, Jiamin Qian, Jianwei Mu, Ruiying Liu, Xiaoya Li, Xueping Li, Chen Jin, Lu Feng, Mingqing Zhang, Yu Pang

**Affiliations:** 1 TEDA Institute of Biological Sciences and Biotechnology, Nankai University, Tianjin, China; 2 Department of Colorectal Surgery, Tianjin Union Medical Center, The First Affiliated Hospital of Nankai University, Tianjin, China; 3 Tianjin Institute of Coloproctology, Tianjin, China; 4 Nankai International Advanced Research Institute, Nankai University Shenzhen, Shenzhen, China; 5 School of Life Sciences, Tianjin University, Tianjin, China

**Keywords:** molecular biology, microbiology

## Abstract

Urinary tract infections (UTIs), primarily caused by uropathogenic *Escherichia coli* (UPEC), are a significant public health problem. UPEC relies on type 1 fimbria to invade bladder epithelial cells (BECs), which is a crucial step in the successful establishment of UTIs. In this study, we found that a two-component system, RstAB, was required for UPEC CFT073 colonization of the mouse bladder, by contributing to bacterial invasion during acute UTIs. Our results demonstrated that RstAB promoted UPEC invasion of BECs and enhanced virulence by activating type 1 fimbria, with RstA directly binding to *fimS* to facilitate its phase-ON orientation. This study identified a pathway regulating type 1 fimbria activation in UPEC CFT073, providing mechanistic insights into its virulence and a potential strategy for UTI treatment.

## Highlights

• UPEC TCS RstAB is critical for CFT073 bladder colonization by promoting its invasion
• RstAB upregulates type 1 fimbria expression
• RstA directly binds to *fimS* and promotes its phase-ON inversion
• RstAB is activated in response to Fe^2+^ *in vitro*

## Graphical abstract

**Figure:**
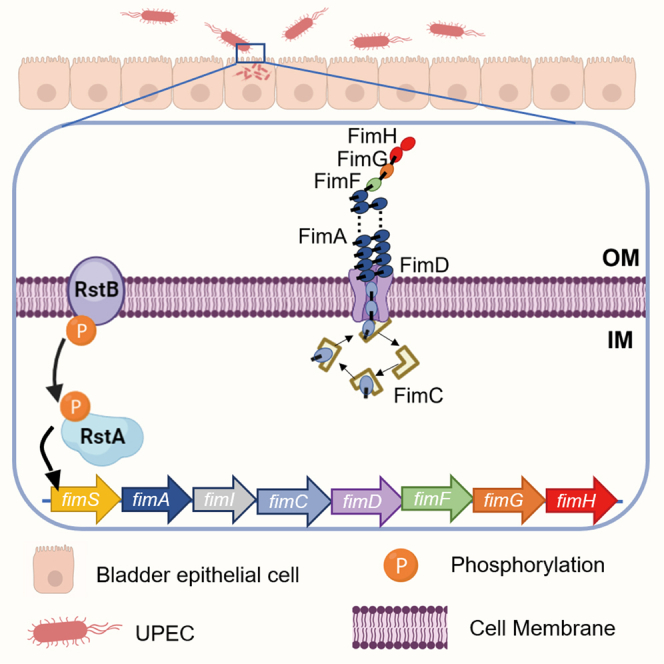

## Introduction

Urinary tract infection (UTI) is one of the most prevalent bacterial infections affecting humans, with an estimated 150 million people affected worldwide every year.^1^ UTIs affect individuals of all ages and sexes, with a significantly higher prevalence among women. Certain populations demonstrate particular susceptibility, including pediatric and geriatric patients, immunocompromised individuals, and hospitalized patients.^2^ UPEC is the predominant etiological agent of UTIs, responsible for approximately 85% of cases. UPEC primarily induces bladder (cystitis) and kidney (pyelonephritis) infections, leading to localized inflammation and clinical symptoms.^3^ In extreme cases, UPEC can progress to sepsis.^4^ Despite appropriate antibiotic therapies, UTIs have an extremely high rate of recurrence.^5^

The pathogenesis of UTIs begins when UPEC enters the urinary tract.^6^ To counteract the host defense of urinary bacterial clearance, UPEC gains a foothold in the bladder by binding mannose residues on bladder epithelial cells (BECs) through FimH, a mannose-binding adhesin located at the tip of the type 1 fimbria.^7^^,^^8^ To obtain better survival sites, UPEC escape into the BEC cytosol where they rapidly replicate and form intracellular bacterial communities (IBCs).^9^ As IBCs mature, bacteria within IBCs efflux to the bladder lumen and invade neighboring naive BECs, creating IBC formation cycles.^10^^,^^11^ Through this cyclical IBC formation strategy, UPEC achieves dual evasion of host immune defenses and antibiotic activity, ultimately promoting recurrent UTI.^12^^,^^13^

Type 1 fimbria are essential for UPEC invasion of BECs.^14^ Compared to wild-type (WT) UPEC UTI89, the type 1 fimbria mutant has a significantly reduced ability to invade the bladder in the C3H/HeN mouse model.^15^ The *fimH* mutant of NU14 is noninvasive in human BECs 5637.^16^ Type 1 fimbriae consist of a major rod subunit, FimA; two minor subunits, FimF and FimG, which form the distal tip of the fibrillum; and FimH, an adhesin.^17^ FimH, which is located at the terminus of the type 1 fimbria, consists of an N-terminal mannoside-binding lectin structural domain (FimHL) and a C-terminal pilocarpine protein structural domain (FimHP).^18^ FimH facilitates UPEC adhesion by specifically recognizing terminal mannose residues (uroplakin 1α as well as β1 and α3 integrins) on glycoproteins that are abundantly expressed on the surface of BECs.^14^^,^^19^^,^^20^

The expression of type 1 fimbria is controlled by an invertible element (314 bp), *fimS*, which is also known as a phase switch.^21^ This element regulates the transcription of the structural subunit genes (*fimA*) and other auxiliary genes (*fimAICDFGH*) required for type 1 fimbria assembly.^22^ When the switch is in the “OFF” orientation, *fimS* is aligned to *fimA* and the expression of type 1 fimbria is arrested, whereas when the phase switch is in the “ON” orientation, *fimS* is inverted to *fimA* with the ensuing transcription of type 1 fimbria.^23^ Inversion of *fimS* is mediated by the recombinases FimE and FmB.^24^ The recombinase FimE primarily facilitates ON-to-OFF switching, whereas FimB can switch in either directions.^25^ The phase orientation of *fimS* is regulated by recombination directionality factors, such as histone-like nucleoid structuring (H-NS), leucine-responsive proteins (LRPs), and integration host factors (IHF). These factors directly bind to *fimS* and facilitate DNA relaxation, or indirectly regulate *fimB* and *fimE*, thereby controlling the phase inversion of *fimS*.^26^^,^^27^^,^^28^

The two-component system (TCS) is an important regulatory mechanism that enables bacteria to adapt to dynamic environments.^29^ Several TCSs have been reported to be involved in the pathogenesis of UPEC. The KguSR TCS facilitates UPEC colonization of mouse urinary tracts in response to the presence of the metabolite α-ketoglutarate.^30^ Mutants of *kguSR* significantly reduce bacterial colonization in the bladder and kidneys of mice.^31^ In a macaque cystitis model, the BarA/UvrY TCS is a determinant of virulence, and disruption of BarA/UvrY impairs the ability of bacteria to switch between gluconeogenic and glycolytic carbon sources, thereby reducing their virulence and environmental adaptability.^32^ The mutation of either *barA* or *uvrY* reduces the production of inflammatory cytokines (TNF-a, IL-6, and IL-8) in UPEC CFT073-infected HK-2 epithelial cells.^33^ OrhKR TCS of CFT073 activates the expression of oxidative stress resistance proteins and hemolysin (encoded by *hlyA*) in response to H_2_O_2_, which protects UPEC from intracellular oxidative stress and simultaneously promotes hemolysin-mediated pyroptotic cell death, leading to host tissue damage.^34^ The CpxRA TCS is required for the fitness and virulence of two reference UPEC strains, UTI89 and CFT073, in mouse UTI and zebrafish infection models.^35^^,^^36^ CpxRA regulates the expression of P pili (filamentous adhesion organelle), which promotes bacterial interactions with host kidney cells and thus influences bacterial virulence.^37^^,^^38^

TCS has been shown to regulate type 1 fimbrial expression in UPEC. PhoBR TCS is induced by deleting the *pst* system or phosphate starvation. PhoBR reduces UPEC virulence in a UTI mouse model by activating YaiC, increasing c-di-GMP accumulation, repressing the *fim* operon, and inhibiting type 1 fimbrial expression.^39^^,^^40^ The EnvZ/OmpR TCS represses *fimB* transcription, indirectly downregulating type 1 fimbrial expression under acidic and high-osmolality conditions *in vitro.*^41^ Mutants of *envZ*/*ompR* have a significantly lower survival rate than the NU149 WT strain in mouse bladder and kidney colonization assays.^42^ However, whether EnvZ/OmpR affects bacterial virulence by regulating type 1 fimbrial expression remains unclear. The expression of *qseB* leads to the downregulation of type 1 fimbrial expression, which significantly impairs bacterial colonization and IBC formation.^43^^,^^44^ QseBC and PmrAB co-regulate *qseB* expression.^45^ However, the mechanisms through which QseBC and PmrAB regulate type 1 fimbrial expression remain unclear. Our previous study found that a new TCS, GrpPQ, activated type 1 fimbrial expression in UPEC CFT073, with GrpQ directly binding to *fimS* and facilitating its phase-ON state, thereby increasing the invasion of BECs by UPEC and contributing to its virulence.^46^

Recently, the functional TCS RstAB was reported to regulate bacterial virulence in *Vibrio alginolyticus* (*V. alginolyticus*),^47^ *Vibrio cholerae* (*V. cholerae*),^48^ *Salmonella typhimurium* (*S. typhimurium*),^49^^,^^50^ adherent-invasive *Escherichia coli* (AIEC),^51^ and avian pathogenic *Escherichia coli* (APEC).^52^ The biological processes mediated by RstAB are diverse and include cell membrane formation, motility, adaptation to acid-tolerant environments, hemolysis, chemotaxis, and flagella formation.^47^^,^^49^^,^^50^^,^^51^ However, whether RstAB contributes to UPEC virulence remains unclear.

In this study, we found that RstAB contributes to UPEC virulence in acute UTI by comparing bladder colonization by *rstAB* mutant and WT strains in a mouse UTI model. Mouse bladder and cell line invasion experiments showed that RstAB is required for UPEC invasion of BECs. RNA sequencing (RNA-seq) data showed that the expression of type 1 fimbrial genes was significantly downregulated in the *rstAB* deletion mutant (Δ*rstAB)* compared with that in WT cultured in Luria-Bertani (LB) medium. Quantitative real-time PCR (real-time qPCR) confirmed that the mRNA levels of type 1 fimbrial genes were significantly downregulated in Δ*rstAB* compared with those in the WT *in vitro* and *in vivo*. Electrophoretic mobility shift assay (EMSA) showed that RstA directly binds to *fimS*. The assay for *fimS* orientation showed that RstAB facilitates the transition of *fimS* from the OFF phase to the ON phase. Western blot and hemagglutination assay (HA) results showed that RstAB activated type 1 fimbrial expression, resulting in enhanced FimH production and surface expression. In this study, we demonstrated that RstAB contributes to bacterial virulence in mouse bladders by facilitating UPEC invasion of BECs. Mechanistically, RstA directly binds to *fimS* to facilitate the ON phase variation, thereby activating the expression of type 1 fimbria. Our work illustrates the virulence-related function of RstAB in UPEC and provides a promising approach for UTI treatment and recurrence prevention.

## Results

### RstAB influences UPEC CFT073 virulence

**Figure 1:**
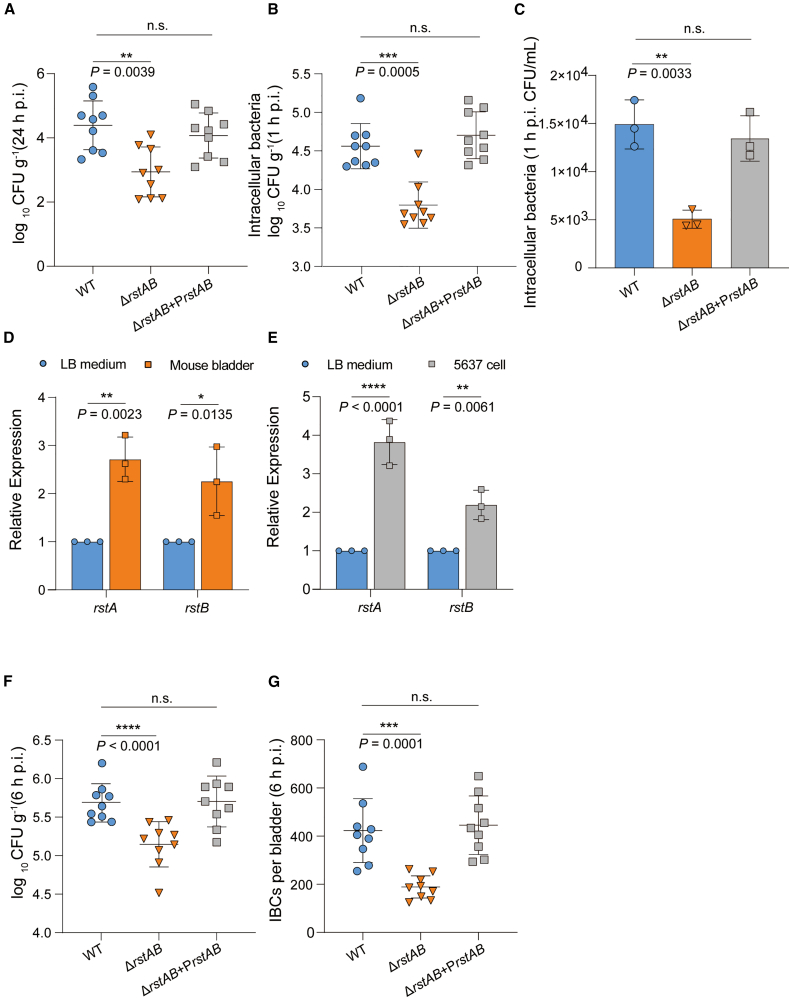
*rstAB* contributes to UPEC CFT073 virulence by promoting its invasion of BECs (A) Total bacterial titers of UPEC CFT073 in the bladders of BALB/c mice transurethrally infected with WT, Δ*rstAB*, or Δ*rstAB* + P*rstAB* at 24 hpi (*n* = 9 mice). (B) Intracellular bacterial titers of UPEC CFT073 in the bladders of BALB/c mice transurethrally infected with WT, Δ*rstAB*, or Δ*rstAB* + P*rstAB* at 1 hpi (*n* = 9 mice). (C) Intracellular bacterial titers of WT, Δ*rstAB*, or Δ*rstAB* + P*rstAB* in 5637 cells at 1 hpi (*n* = 3). (D) Fold changes in *rstA* and *rstB* mRNA levels in WT-infected BALB/c mouse bladders at 1 hpi compared to that of WT cultured in LB medium (*n* = 3). (E) Fold changes in *rstA* and *rstB* mRNA levels in WT-infected 5637 cells at 1 hpi (*n* = 3) compared to that of WT cultured in LB medium. (F) Total bacterial titers of UPEC CFT073 in the bladders of BALB/c mice transurethrally infected with WT, Δ*rstAB*, or Δ*rstAB* + P*rstAB* at 6 hpi (*n* = 9 mice). (G) IBC enumeration in C3H/HeN mouse bladders transurethrally infected with WT, Δ*rstAB*, or Δ*rstAB* + P*rstAB* at 6 hpi determined using confocal microscopy (*n* = 9 mice). Data were obtained from three independent experiments and presented as mean ± SD. Significance was determined using two-tailed Mann-Whitney *U* test (A, B, F, and G) and two-tailed unpaired Student’s *t* test (C, D, and E). Significance was indicated by *p* value. ∗*p* ≤ 0.05, ∗∗*p* ≤ 0.01, ∗∗∗*p* ≤ 0.001, ∗∗∗∗*p* ＜0.0001; n.s. represents no significant difference. See also Figures S1 and S2.

To investigate whether *rstAB* impacts UPEC virulence, we constructed an *rstAB* deletion mutant (Δ*rstAB*) and a back-complementary strain (Δ*rstAB* + P*rstAB*). The colony-forming units (CFUs) in the bladders of Δ*rstAB*-infected mice were 1.5-log-fold lower than those of WT, whereas Δ*rstAB* + P*rstAB* restored the CFUs to a level similar to that of the WT (Figure 1A). This result indicated that a lack of *rstAB* decreased the colonization of UPEC CFT073 in mouse bladders. To exclude the possibility that the reduced bacterial colonization ability of the *rstAB* deletion mutant was due to its growth defect, we measured the growth curves of the WT, Δ*rstAB*, and Δ*rstAB* + P*rstAB* strains. The results showed that there was no significant growth difference between the WT, Δ*rstAB*, and Δ*rstAB* + P*rstAB* strains in RPMI 1640 as well as in the LB medium (Figure S1). Taken together, these results suggest that *rstAB* promotes UPEC colonization of mouse bladders during acute UTI, thereby contributing to UPEC virulence.

### RstAB promotes UPEC CFT073 to invade BECs

BEC invasion is the first step in the pathogenesis of UPEC infection.^14^ To verify whether *rstAB* affected UPEC invasion of BECs in the mouse bladders, we performed mouse bladder invasion experiments with WT, Δ*rstAB*, or Δ*rstAB* + P*rstAB* at 1 hour post-infection (hpi), the point at which UPEC invades BECs.^15^ The intracellular CFUs of the Δ*rstAB-*infected mouse bladders was 1.2-log-fold lower than those of the WT, whereas Δ*rstAB* + P*rstAB* restored the intracellular CFUs to a level similar to that of the WT (Figure 1B). This result indicates that *rstAB* increases UPEC invasion of BECs *in vivo*.

Additionally, we have investigated the role of *rstAB* in mediating the invasion of cultured BEC 5637. We infected 5637 cells with WT, Δ*rstAB*, or Δ*rstAB* + P*rstAB* for 30 min. After gentamicin treatment for an additional 30 min, intracellular bacterial CFUs were counted. The results showed that the intracellular CFUs of Δ*rstAB*-infected 5637 cells were significantly reduced by 2.9-fold compared to those of WT and that Δ*rstAB* + P*rstAB* restored the CFUs to a similar level to that of WT (Figure 1C). Collectively, these results indicated that *rstAB* enhances UPEC CFT073 invasion of BECs, both *in vitro* and *in vivo*.

To exclude that the reduced intracellular CFUs were due to the host cell loss, we performed lactatedehydrogenase (LDH) assays on 5637 cells infected with WT, Δ*rstAB*, or Δ*rstAB* + P*rstAB* strains at 1 hpi. The result showed that the LDH release of WT-, Δ*rstAB-*, and Δ*rstAB* + P*rstAB*-infected 5637 cells was at a similar level, indicating that *rstAB* mutant does not affect the 5637 cell death (Figure S2A).

The expression levels of *rstA* and *rstB* were measured in WT-infected mouse bladders and 5637 cells. The results showed that the expressions of *rstA* and *rstB* were significantly upregulated in WT-infected mouse bladders and 5637 cells at 1 hpi compared with that in WT cultured in LB medium (Figures 1D and 1E), indicating that the expression of *rstA* and *rstB* was upregulated during UPEC CFT073 invasion of BECs. The amount of RstA and RstB production in UPEC-infected mouse bladder was confirmed using western blotting. Results showed that post-invasion into mouse bladder, RstA-FLAG protein production increased 6.2-fold, and RstB-Flag protein production increased 7.1-fold compared to LB medium (Figures S2B and S2C). These findings indicate that the expression and production of RstA and RstB are upregulated during host invasion.

### RstAB contributes to IBC formation

UPEC form IBCs in the cytoplasm after invading BECs.^10^^,^^11^ We investigated whether *rstAB*-enhanced UPEC invasion resulted in increased IBC formation. IBC formation in mouse bladders is relatively synchronous and highest at 6 hpi.^13^ BALB/c mice were transurethrally infected with WT, Δ*rstAB*, or Δ*rstAB* + P*rstAB*, and the bacterial CFUs in the bladders were determined at 6 hpi. The results showed that bacterial CFUs in Δ*rstAB*-infected mouse bladders were 1.1-log-fold lower than those in WT-infected mouse bladders, whereas Δ*rstAB* + P*rstAB* restored the bacterial CFUs in mouse bladders to a level similar to that of WT (Figure 1F). This result indicates that *rstAB* promotes IBC formation in UPEC-colonized mouse bladders.

The morphology of IBCs was directly observed using wheat germ agglutinin staining.^53^ We measured the number of IBCs per bladder in C3H/HeN mice transurethrally infected with WT-GFP, Δ*rstAB*-GFP, or Δ*rstAB* + P*rstAB*-GFP at 6 hpi (Figure 1G). The results showed that the number of IBCs per bladder of Δ*rstAB*-infected mice was significantly reduced by 2.2-fold compared with that of WT-infected mice; the IBC number was restored in the bladder of Δ*rstAB* + P*rstAB*-infected mice. These results indicate that *rstAB* increases the number of UPEC CFT073 in mouse bladders. The results of IBC formation were consistent with those of bacterial invasion, suggesting that *rstAB* contributes to UPEC CFT073 virulence by promoting BEC invasion.

### Analysis of downstream gene expression regulated by RstAB using transcriptomics

**Figure 2:**
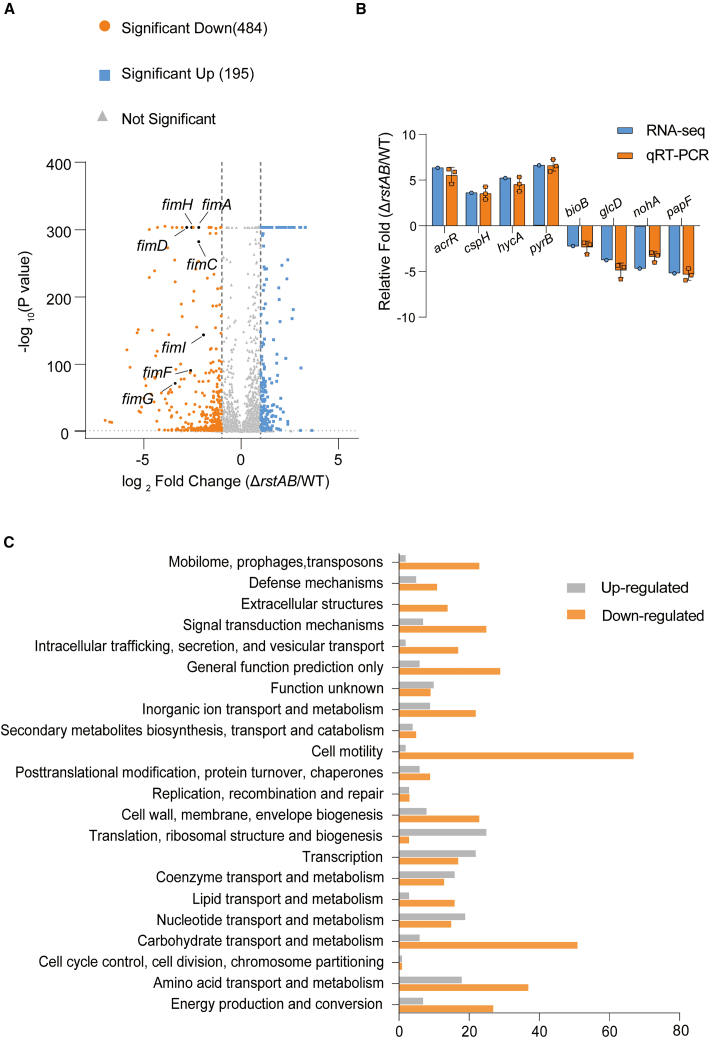
Transcriptome analysis of the downstream gene of RstAB (A) Volcano plot showing DEGs in the transcriptome of Δ*rstAB* in the same static culture of LB medium compared to that of WT. Each dot denotes one gene. Blue dots represent upreguled genes (*p* ≤ 0.05, log_2_foldchange ≥1) and orange dots represent downreguled genes (*p* ≤ 0.05, log_2_foldchange ≤ −1). (B) Real-time qPCR analysis of the expression of 8 randomly selected differentially expressed genes captured from the RNA-seq profile. Data were obtained from three independent experiments and presented as mean ± SD. (C) COG analysis of DEGs in the transcriptome of Δ*rstAB* in the same static culture of LB medium compared to that of WT.

To identify the downstream genes regulated by RstAB in UPEC CFT073, we performed a high-throughput RNA sequencing experiment on WT and Δ*rstAB* statically cultured in LB medium (PRJNA1277113). A total of 679 genes showed differential expression between WT and Δ*rstAB* based on the parameters *p* value <0.05 and |log2 foldchange| ≥ 1. Of these, 484 genes were downregulated and 195 genes were upregulated in Δ*rstAB* compared with those of the WT (Figure 2A). To ensure the stable validity of the transcriptomic data, we randomly selected five downregulated genes and five upregulated genes and verified their expression using real-time qPCR. The real-time qPCR results correlated well with the transcriptomic data (Figure 2B), indicating that our RNA-seq data were reproducible and of good quality for further analysis. Differentially expressed genes (DEGs) in the WT and Δ*rstAB* groups were classified using the NCBI clusters of orthologous groups (COG) functional category annotation system. The COG categories cell motility, carbohydrate transport and metabolism, amino acid transport and metabolism, and signal transduction mechanisms were significantly downregulated in the Δ*rstAB* group. The COG categories of translation, ribosomal structure and biogenesis, transcription, nucleotide transport and metabolism, and coenzyme transport and metabolism were significantly upregulated in Δ*rstAB* group (Figure 2C).

Notably, in our RNA-seq profile, the expression of all seven genes (*fimACDFGHI*) encoding type 1 fimbria were downregulated (3.8- to 10.5-fold) in Δ*rstAB* compared with that in the WT. Type 1 fimbria are essential for UPEC invasion; therefore, we speculated that *rstAB* promotes UPEC CFT073 invasion by activating type 1 fimbria.

### RstAB activates the expression and production of type 1 fimbria

**Figure 3:**
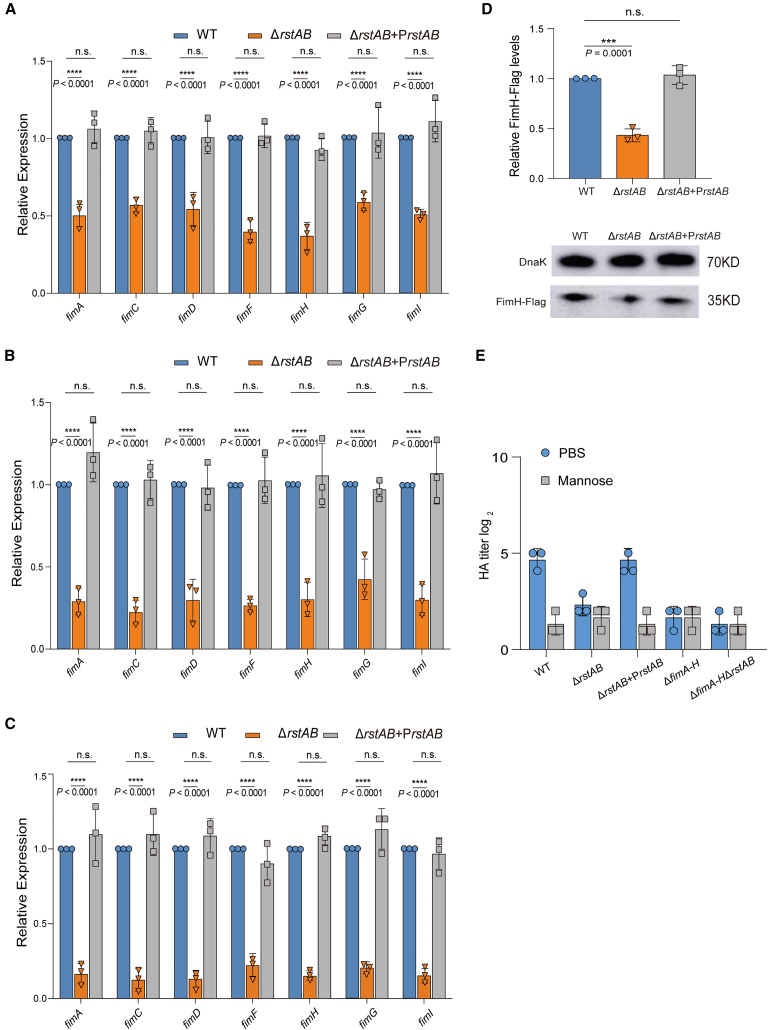
RstAB activates the expression and production of type 1 fimbria (A) Real-time qPCR analyses of the mRNA levels of *fimA*, *fimC*, *fimD*, *fimF*, *fimH*, *fimG*, and *fimI* in WT, Δ*rstAB*, or Δ*rstAB* + P*rstAB* statically cultured in LB medium for 12 h. (B) Real-time qPCR analyses of the mRNA levels of *fimA*, *fimC*, *fimD*, *fimF*, *fimH*, *fimG*, and *fimI* in WT-, Δ*rstAB-*, or Δ*rstAB* + P*rstAB*-infected 5637 cells at 1 hpi. (C) Real-time qPCR analyses of the mRNA levels of *fimA*, *fimC*, *fimD*, *fimF*, *fimH*, *fimG*, and *fimI* in WT-, Δ*rstAB*-, or Δ*rstAB* + P*rstAB*-infected mouse bladders at 1 hpi. (D) Quantitative analysis of FimH protein levels in WT, Δ*rstAB*, or Δ*rstAB* + P*rstAB* statically grown in LB medium at 37°C overnight (top). DnaK served as a loading control. Representative image (bottom) from three independent experiments. (E) HA assays of the production of type 1 fimbria in WT, Δ*rstAB*, Δ*rstAB* + P*rstAB*, Δ*fimA-H*, or Δ*fimAH*Δ*rstAB* in the presence or absence of 3% mannose. Data were obtained from three independent experiments and presented as mean ± SD. Significance was determined by two-tailed unpaired Student’s *t* test (A–D) and two-way analysis of variance (E). Significance was indicated by a *p* value. ∗∗∗*p* ≤ 0.001, ∗∗∗∗*p* ＜0.0001; n.s. no significant difference. See also Figures S3 and S5.

To investigate whether *rstAB* affects the expression of type 1 fimbria, we extracted RNA from WT, Δ*rstAB*, or Δ*rstAB* + P*rstAB* statically cultured in LB medium and analyzed it using real-time qPCR. The result showed that the mRNA levels of *fimACDFGHI* were significantly downregulated by 1.7- to 2.5-fold in Δ*rstAB* compared with those in the WT (Figure 3A), indicating that RstAB positively regulated type 1 fimbrial expression *in vitro*. Furthermore, we analyzed the mRNA levels of *fimACDFGHI* in WT-, Δ*rstAB-*, and Δ*rstAB* + P*rstAB-*infected 5637 cells or mouse bladders 1 h after infection. The results showed that the mRNA levels of *fimACDFGHI* were significantly downregulated by 2.3- to 4.4-fold in Δ*rstAB-*infected 5637 cells compared with those in WT mice (Figure 3B), indicating that RstAB positively regulated type 1 fimbrial expression in UPEC CFT073-infected BECs. The results showed that the mRNA levels of *fimACDFGHI* were significantly downregulated by 4.5- to 8.0-fold in Δ*rstAB-*infected mouse bladders compared with those in WT mice at 1 hpi (Figure 3C), indicating that RstAB positively regulated type 1 fimbrial expression in UPEC CFT073-infected mouse bladders. Meanwhile, the mRNA levels of *fimACDFGHI* in Δ*rstAB* + P*rstAB* were restored to WT levels in UPEC CFT073-infected 5637 cells and mouse bladders. These results indicated that RstAB positively regulates the expression of type 1 fimbria both *in vitro* and *in vivo*.

FimH at the tip of the type 1 fimbria recognizes molecular receptors on the surface of BECs and promotes UPEC invasion of BECs.^7^^,^^8^ Next, we examined whether RstAB-regulated type 1 fimbrial expression increased FimH production. We performed western blotting to analyze the FimH protein levels in WT-FimH-FLAG, Δ*rstAB*-FimH-FLAG, or Δ*rstAB* + P*rstAB*-FimH-FLAG cultured in LB medium. The results showed that the production of FimH in Δ*rstAB* was 2.3-fold lower than that in the WT (Figure 3D), whereas Δ*rstAB* + P*rstAB* restored the production of FimH to a similar level to the WT. This result indicated that RstAB-mediated activation of type 1 fimbrial expression increased FimH production.

Next, we investigated whether RstAB increases the production of FimH, leading to the surface expression of type 1 fimbria using HA.^54^ The HA titer was represented by the highest dilution that showed positive agglutination. The results showed that the HA titer of Δ*rstAB* was significantly lower than that of WT and Δ*rstAB* + P*rstAB* restored the decreased HA titer to a level similar to that of WT, indicating that RstAB promotes the surface expression of type 1 fimbria (Figure S5). As a negative control, we constructed a Δ*fimA-H* mutant strain,^55^ which lacked type 1 fimbrial expression and almost no hemagglutination, as well as a double mutant strain Δ*fimA-H*Δ*rstAB*, which lacked *fimA-H* and *rstAB* gene expression. The HA titer of Δ*fimA-H*Δ*rstAB* was identical to that of Δ*rstAB* and Δ*fimA-H*, indicating that the contribution of RstAB on surface formation of type 1 fimbria was dependent on *fimA-H*. In the presence of 3% mannose (a competitive inhibitor of type 1 fimbria-mediated adhesion), the difference in HA titers between WT and Δ*rstAB* was eradicated (Figure 3E). These results suggest that RstAB promotes the production of type 1 fimbria on bacterial surfaces.

### RstAB directly binds to *fimS* to facilitate its inversion to phase-ON orientation

**Figure 4:**
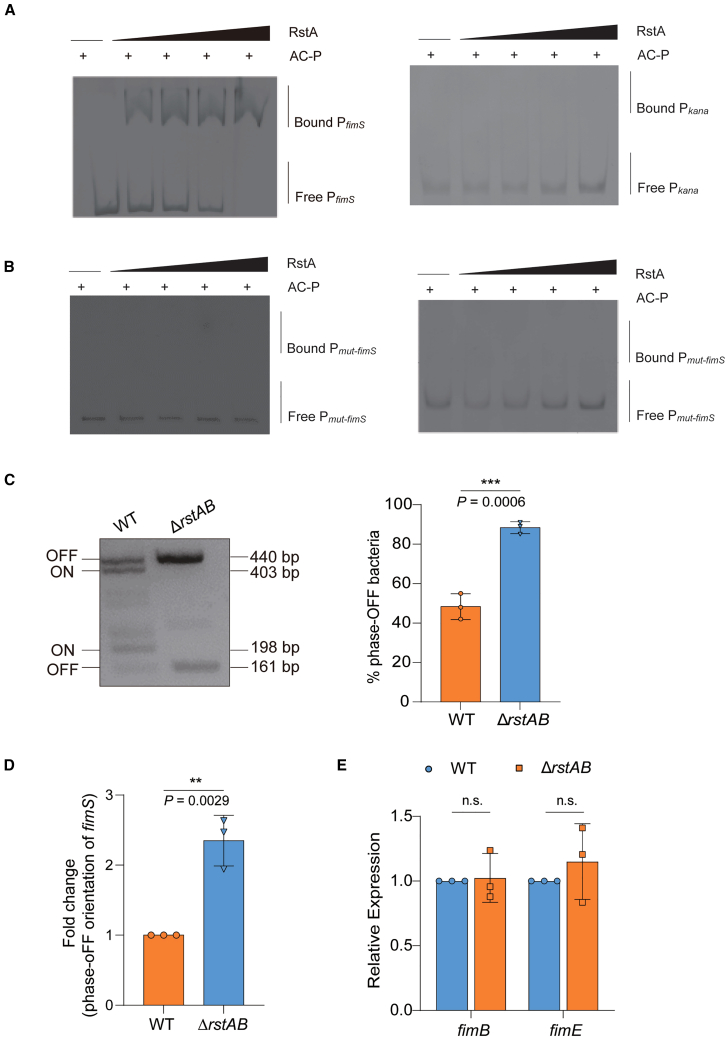
RstAB directly binds to *fimS* to facilitate its inversion to phase-ON orientation (A) EMSA of the binding of purified RstA to *fimS* and the kanamycin promoter (*kana*). PCR products were added to the reaction mixtures at 40 ng each. RstA protein was added to the reaction buffer in each assay in lanes 1–5 at 0, 0.25, 0.5, 1, and 2 μM, respectively. (B) EMSA of the binding of purified RstA to mut-*fimS* and the kanamycin promoter (*kana*). PCR products were added to the reaction mixtures at 40 ng each. RstA protein was added to the reaction buffer in each assay in lanes 1–5 at 0, 0.25, 0.5, 1, and 2 μM, respectively. (C) Quantification of the percentage of bacteria with *fimS* in the phase-OFF orientation. (D) qPCR analysis the number of OFF- orientation amplification products of Δ*rstAB* relative to WT. (E) Real-time qPCR analysis of the mRNA levels of *fimB* and *fimE* in Δ*rstAB* compared to that of WT. The results are representative of three biological replicate experiments (A and B). Data were obtained from three independent experiments and are presented as mean ± SD (C–E). *p* values were determined using two-tailed Student’s *t* test (C–E). ∗∗*p* ≤ 0.01, ∗∗∗*p* ≤ 0.001; n.s., no significant difference.

The RstA-binding site (RstA box) is composed of the consensus sequence TACATNTNGTTACA, where “N” stands for any nucleotide.^56^ Using a search pattern tool (http://genolist.pasteur.fr/SubtiList), a similar sequence (TAAAGATAACTATA) was identified in *fimS*. Next, we investigated whether RstA binds directly to *fimS in vitro*. The EMSA results showed that *fimS* DNA fragments exhibited slow-migrating bands as the concentration of the RstA protein increased, whereas no migrating bands were observed for the negative control (kanamycin cassette fragment) (Figure 4A), indicating that RstA directly binds to *fimS in vitro*. To determine whether RstA binds to *fimS* dependent on the RstA box in UPEC CFT073, we mutated the 14 bases in the *fimS* fragment to obtain the *fimS*-mut fragment (5ʹ- CGGGAGCGGTCGCG-3ʹ). The EMSA results showed that mutation of this RstA-binding site completely abolished the binding of RstA to *fimS* (Figure 4B), indicating that RstA specifically binds to the RstA box on *fimS*.

Next, we analyzed whether RstA binds to *fimS* and alters its phase. Based on the method of Stentebjerg-Olesen et al.,^57^ the DNA fragments of *fimS* were digested with the restriction endonuclease SnaBI. Since SnaBI cleaves *fimS* in an asymmetric position, the proportion of bacteria in the “phase-ON” or “OFF” state can be calculated based on the intensity of the bands of the cleaved fragments. The results showed that 88.3% of *fimS* proteins were in the phase-OFF orientation in Δ*rstAB*, while there was 48.3% in the WT. This indicated that RstAB facilitated the transition of *fimS* from OFF to ON (Figure 4C). Additionally, we performed qPCR using phase-OFF-oriented RT primers to determine the *fimS* inversion state.^40^ The results showed that in the same 50-ng genomic template of WT and Δ*rstAB*, the number of phase-OFF orientation amplification products of Δ*rstAB* was 2.3-fold higher than that in the WT (Figure 4D). This indicates that RstAB facilitates the transition of *fimS* from the OFF to the ON phase. *fimS* phase inversion is also mediated by the recombinant enzymes FimE and FimB.^24^ We investigated whether RstAB affects *fimS* phase inversion by influencing the expression of *fimE* or *fimB*. We performed real-time qPCR to detect the expression of *fimE* and *fimB* in WT and Δ*rstAB* cultured in LB medium. The results showed that the mRNA levels of *fimB* and *fimE* in Δ*rstAB* were similar to those in the WT (Figure 4E), indicating that RstAB does not regulate the expression of type 1 fimbria through FimB or FimE. Collectively, these data suggest that RstAB activates type 1 fimbrial expression via the direct binding of RstA to *fimS* to facilitate its inversion to the phase ON orientation.

To further demonstrate whether the binding site on *fimS* for the RtsA protein is dependent on RstA-box, we used β-galactosidase assays to measure the transcriptional activity of *fimS* and *fimS-mut* (lack the RstA box) to confirm whether the RstA-binding site is on *fimS*. Results showed that the transcriptional activity of *fimS* was significantly higher than that of *fimS*-mut in WT. In Δ*rstAB*, the transcriptional activity of *fimS* was similar to that of *fimS*-mut, and both were substantially lower than that of *fimS* in WT (Figure S3). This result suggests that RstA specifically binds to the RstA box on *fimS*.

### RstAB promotes UPEC invasion by activating the expression of type 1 fimbria

**Figure 5:**
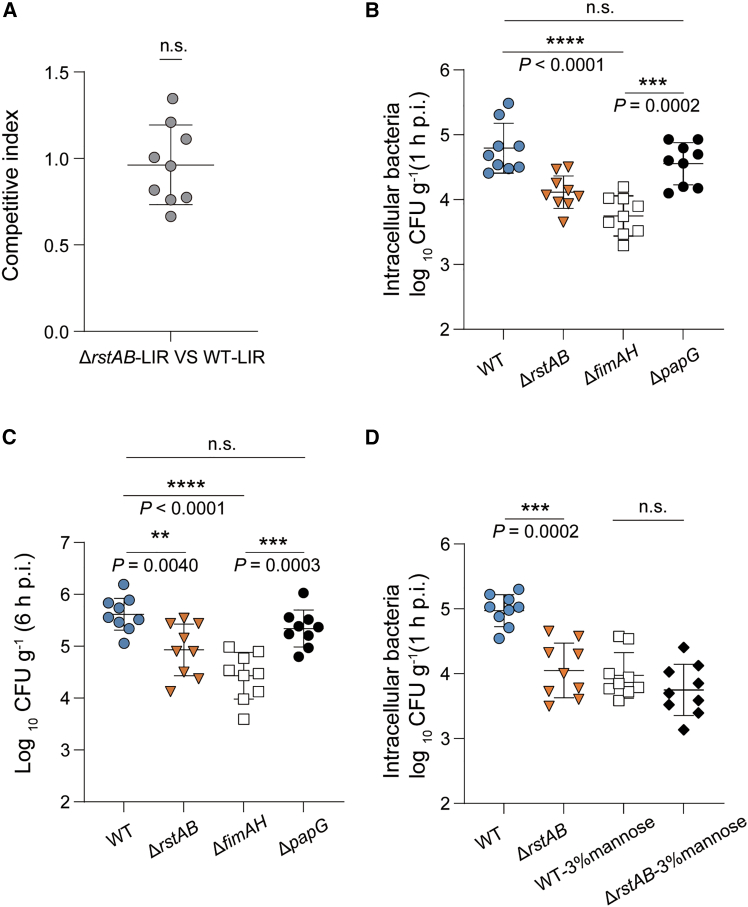
RstAB promotes UPEC invasion by activating the expression of type 1 fimbria (A) Competitive index of Δ*rstAB*-LIR versus WT-LIR in BALB/c mouse bladders at 1 hpi (*n* = 9 mice). (B) Intracellular bacterial titers of UPEC CFT073 in the bladders of BALB/c mice transurethrally infected with WT, Δ*rstAB*, Δ*fimAH*, or Δ*papG* at 1 hpi (*n* = 9 mice). (C) Total bacterial titers of UPEC CFT073 in the bladders of BALB/c mice transurethrally infected with WT, Δ*rstAB*, Δ*fimAH*, or Δ*papG* at 6 hpi (*n* = 9 mice). (D) Bacterial titers in the bladders of BALB/c mice infected transurethrally with WT or Δ*rstAB* strains, with or without 3% mannose at 1 hpi (*n* = 9 mice). Data were obtained from three independent experiments and presented as mean ± SD. *p* values were determined using two-tailed Wilcoxon signal-rank test (A) or two tailed Mann-Whitney *U* test (B–D). Significance was indicated by *p* value. ∗∗∗*p* ≤ 0.001, ∗∗∗∗*p* ＜ 0.0001, n.s. represents no significant difference. See also Figure S4.

To verify that the invasion of BECs facilitated by RstAB is dependent on type 1 fimbria, we constructed *fimS* locked-phase-ON in the WT and Δ*rstAB* background. The *fimS* inversion was blocked in the mutant bacteria, thus allowing the *fimS* promoter progenitor to continuously phase-lock in the phase ON orientation,^43^ yielding the WT-LIR and Δ*rstAB*-LIR. WT-LIR and Δ*rstAB*-LIR were co-infected in mice at a ratio of 1:1 for 1 h, and the colonization ability of these two strains in mouse bladders was compared by calculating the competition index (CI). The results showed that the CI of Δ*rstAB*-LIR versus that of WT-LIR averaged approximately 0.96 and was not statistically significant (Figure 5A), indicating that Δ*rstAB*-LIR and WT-LIR have similar invasion abilities, suggesting that the *rstAB* mutation did not confer additional invasion defects in the *fimS* phase-locked-on background. These results demonstrate that RstAB promotes invasion of the UPEC strain CFT073 into BECs by activating type 1 fimbrial expression.

To determine whether the contribution of *rstAB* to UPEC invasion capacity is entirely attributable to type 1 fimbria. We performed mouse bladder invasion experiments with WT, Δ*rstAB*, or Δ*fimA-H* at 1 hpi. Results showed that the colonization capability of Δ*fimA-H* was lower than that of Δ*rstAB*, but both were significantly decreased compared to WT. This result indicates that RstAB contributes partially to the type 1 fimbria-dependent invasion (Figure 5B).

To verify whether the reduced virulence of Δ*rstAB* depends on FimH *in vivo*, we used D-mannose to block the attachment of type 1 fimbria to BECs and performed the mouse invasion experiment. The results showed that the number of WT that invaded the mouse bladder was lower in the presence of 3% mannose than in its absence, whereas the number of Δ*rstAB* was not affect by the presence or absence of 3% mannose and was lower than that of WT in the absence of 3% mannose (Figure 5D), indicating that RstAB promotes UPEC invasion by activating the expression of type 1 fimbria.

### RstAB is activated in response to Fe^2+^*in vitro*

**Figure 6:**
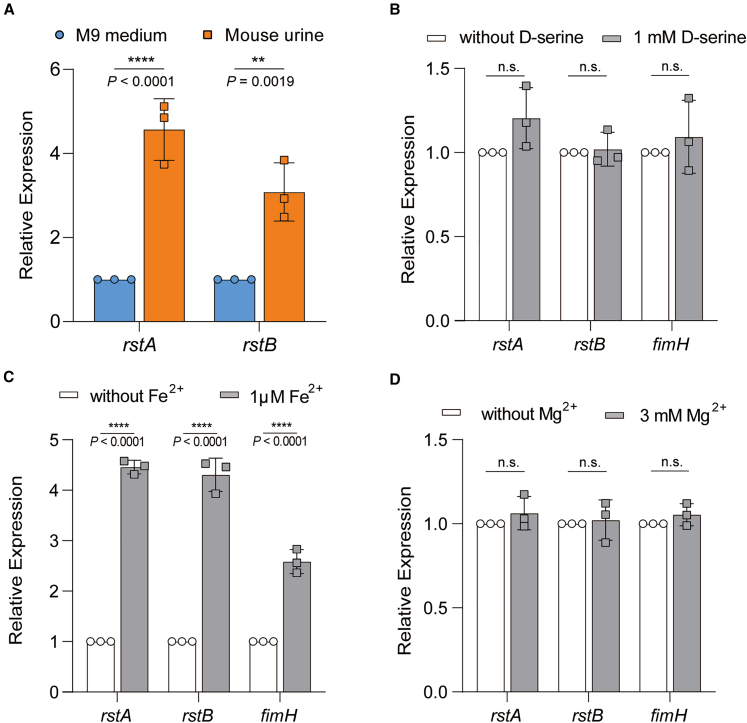
Fe^2+^ is the signal to activate RstAB *in vitro* (A) RstAB TCS is activated by a component in the urine. Real-time qPCR analysis of the mRNA levels of *rstA* or *rstB* in WT cultured in mouse urine compared to that in WT cultured in M9 medium for 1 h. (B–D) Real-time qPCR analyses of the mRNA levels of *rstA*, *rstB*, or *fimH* in WT cultured in M9 medium with the indicated concentrations of D-serine, Fe^2+^, or Mg^2+^. Data were obtained from three independent experiments and presented as mean ± SD. *p* values were determined using two-tailed Student’s *t* test. Significance was indicated by *p* value. ∗∗*p* ≤ 0.01, ∗∗∗∗*p* ＜0.0001, n.s. represents no significant difference.

Considering whether there are cues in urine that induce RstAB expression, we extracted RNA from CFT073 after 1 h of incubation in host urine and performed real-time qPCR analysis. The results showed that the expression of *rstA* and *rstB* was upregulated in CFT073 cultured in mouse urine for 1 h (Figure 6A), indicating that RstAB is activated in host urine. To investigate the upstream signals that activate *rstAB* transcription, we determined the response of RstAB to D-serine (1 mM), Fe^2+^ (1 μM), and Mg^2+^ (3 mM) equivalent to that in host urine.^58^^,^^59^^,^^60^ After 1 h of incubation *in vitro*, D-serine and Mg^2+^ did not affect the mRNA levels of *rstA*, *rstB*, and *fimH* in WT (Figures 6B and 6D), whereas the presence of Fe^2+^ significantly upregulated the mRNA levels of *rstA*, *rstB*, and *fimH* by 4.5-, 4.3-, and 2.6-fold, respectively (Figure 6C). This result indicates that Fe^2+^ at the same concentration in urine can activate RstAB, suggesting that Fe^2+^ is the signal to activate RstAB *in vitro*. However, whether RstAB is activated in response to Fe^2+^ within the host remains to be verified.

## Discussion

UTIs, mainly caused by UPEC, are a major public health problem worldwide. The first stage of the pathogenesis of UTIs is UPEC invasion of BECs via type 1 fimbria, which resists urine expulsion of bacteria.^14^ In this study, we found that the TCS RstAB promotes UPEC CFT073 invasion in BECs, thereby contributing to acute UTI. RNA-seq of WT and Δ*rstAB* cultured in LB medium showed that *rstAB* mutation resulted in downregulation of type 1 fimbrial gene expression. Real-time qPCR confirmed that RstAB upregulated type 1 fimbrial expression *in vitro* in WT-infected 5637 cells and mouse bladders. EMSA, western blotting, and HA results showed that RstA directly binds to the RstA box in *fimS* to activate the expression and production of type 1 fimbria. The results of *fimS* restriction endonuclease section and phase-OFF orientation qPCR assays demonstrated that RstA facilitated the phase inversion of *fimS* from the OFF to ON orientation. In this study, we found that RstAB contributes to UPEC CFT073 virulence by facilitating BEC invasion through the RstAB-mediated activation of type 1 fimbria, in which RstA binds *fimS* and promotes its phase-ON inversion.

The phase variation of *fimS* is directly controlled by multiple recombination directionality factors that bind to *fimS* and regulate its inversion. H-NS directly binds to the DNA segments adjacent to and within the phase-switch region of *fimS*, regulating the phase variation of *fimS* to enhance type 1 fimbrial activity.^26^ Moreover, Lrp and IHF are accessory proteins that affect *fimS* recombination. The expression of *lrp* is repressed by H-NS, which may indirectly modulate *fimS* phase switching.^61^ Lrp binds directly to three different sites (1, 2, and 3) in *fimS* and affects gene recombination.^62^ Lrp binds to high-affinity sites 1 and 2 to increase *fimS* inversion frequency, whereas its binding to the low-affinity site 3 decreases it.^26^^,^^62^^,^^63^ There are two binding sites for IHF: site I adjacent to *fimS* and site II within *fimS*.^63^ IHF binds to sites I and II to regulate *fimS* expression.^63^ Our study identified a novel regulatory site in *fimS* where RstA binds adjacent to the IHF site II, implying that RstA may modulate type 1 fimbrial phase variation in a manner comparable to that of IHF. Unlike PhoB/PhoR and EnvZ/OmpR, which indirectly regulate type 1 fimbria, RstAB and GrpPQ directly regulate type 1 fimbrial expression by binding directly to the *fimS*.

RstAB is an important TCS that contributes to the pathogenesis of several bacterial diseases. RstAB in *V. alginolyticus* responds to environmental signals such as temperature, pH, and starvation.^47^ Mutation of either *rstA* or *rstB* in *V. alginolyticus* impairs motility, hemolysis, and bacterial virulence.^47^ RstAB (CarSR) of *V. cholerae* responds to human defensin 5, which is enriched in the small intestine, and RstA directly binds to the TcpP promoter, upregulating virulence genes to promote bacterial colonization.^48^ RstAB activates FlhA and MglB, which are involved in the modulation of the bacterial motility, chemotaxis, and invasive capacity of *S. typhimurium*.^49^^,^^50^ In AIEC LF82, RstAB responds to acidic pH in macrophages and activates the cell membrane formation gene *csgD* and the acid resistance gene *asr*, thereby promoting its replication in macrophages and intestinal colonization in mice with colitis.^51^ RstAB directly regulates the expression of the acid resistance gene *hdeD*, contributing to the virulence of APEC E058 in chicken macrophages and experimentally infected chickens.^52^ RstAB positively regulates virulence and acid tolerance but negatively regulates biofilm formation in EHEC O157: H7.^64^ Mutation of either *rstA* or *rstB* impairs the secretion of type II secretion system-dependent proteins in *Photobacterium damselae* subsp. *damselae*.^65^ Our findings demonstrate that RstAB contributes to UPEC CFT073 virulence by promoting bacterial invasion of BECs, unveiling the role of RstAB in UPEC pathogenesis.

Several studies have shown that RstA responds to divalent cations. In *S. typhimurium*, RstA is activated by PhoPQ under low Mg^2+^ levels.^49^ The expression of *rstAB* is significantly downregulated in *V. alginolyticus* cultured with heavy metals.^47^ Using RNA-seq, Kong et al. demonstrate that Pb^2+^, Cu^2+^, and Hg^2+^ exposure markedly reduces *rstA* and *rstB* expression. Cu^2+^, Pb^2+^, and Hg^2+^ exposure also downregulates *rstA* expression in *V. alginolyticus*.^66^ The transcription of RstAB decreases with increasing external Ca^2+^ levels, further negatively regulating *vps* gene expression and biofilm formation in *V. cholerae*.^67^ Urine contains abundant divalent cations, such as Ca^2+^ (5 mM), Mg^2+^ (3 mM), and Cu^2+^ (0.17 μM) and is enriched in cationic antimicrobial peptides, such as LL37 (1.8 ng/mg creatinine) and human β-defensin (109.10 ng/mg creatinine).^58^^,^^68^

Analysis of DEGs in the transcriptome revealed that proteins associated with cell adhesion include not only the type 1 fimbria-related genes described in this study but also genes such as *yhhD*, *papF*, *papH*, *papE*, and *fliD*. Furthermore, several genes associated with the bacterial sulfur metabolism pathway exhibited varying degrees of downregulation (Table S3). As well as type 1 fimbria, *pap* is also the most common fimbrial adhesins in *E. coli*. Real-time qPCR confirmed that the mRNA levels of pap-related genes were significantly downregulated in Δ*rstAB* compared with those in the WT *in vitro*. Results showed that the mRNA level of *papG* were significantly downregulated by 3.5-fold in Δ*rstAB* compared to that in WT, indicating that RstAB positively regulates *papG* expression *in vitro* (Figure S4). This result is consistent with transcriptomic data. To verify whether *papG* affects UPEC pathogenicity of BECs in the mouse bladders, we performed mouse bladder invasion and colonization experiments with WT, Δ*rstAB*, or Δ*papG* at 1 and 6 hpi, respectively. The intracellular CFUs of Δ*papG*-infected mouse bladders were comparable to those of WT (Figures 5B and 5C). Meanwhile, relative to Δ*papG*, the invasion and colonization efficiency of Δ*fimA-H* was significantly reduced (Figures 5B and 5C). This finding highlights the dominant role of type 1 fimbria in mediating the UPEC invasion process, relative to P pili. This result indicates that the mutation of *papG* does not impair the ability of UPEC to invade the mouse bladder, which is consistent with a previous report.^69^ Therefore, the regulatory mechanisms between RstAB and type 1 fimbria and Pap pili remain to be further investigation.

### Limitations of the study

This study has several limitations. First, a key limitation of this study is that RNA-seq was performed using bacteria cultured in LB broth under static conditions, which do not faithfully recapitulate the urinary tract microenvironment. The composition of LB medium differs markedly from that of host urine in terms of nutrient availability, osmolality, pH, and ionic content (e.g., divalent cations, urea, and organic metabolites). Consequently, the transcriptional profile of UPEC CFT073 obtained *in vitro* may not fully reflect the gene expression landscape during actual infection. In particular, regulatory pathways that are specifically induced or repressed by urine-specific signals—such as Fe^2+^, D-serine, cationic antimicrobial peptides, or osmotic stress—could have been missed or underestimated in this analysis. Second, whether Fe^2+^ serves as the genuine physiological signal that activates RstAB *in vivo* remains unverified, as no experiments using Fe^2+^ chelators or bacterial Fe^2+^ uptake knockouts were performed to causally link host urinary Fe^2+^ to RstAB activation during infection.

## Resource availability

### Lead contact

Further information and requests for resources and reagents should be directed to and will be fulfilled by the lead contact, Yu Pang (pangyu@nankai.edu.cn).

### Materials availability

Any further requests for resources and reagents should be directed to and will be fulfilled by the lead contact, Dr. Yu Pang (pangyu@nankai.edu.cn). Please note that most reagents could be available upon request after the completion of a materials transfer agreement.

### Data and code availability

• This paper does not report original code.
• Any additional information required to reanalyze the data reported in this paper is available from the lead contact upon request.
• RNA-seq data have been deposited at the NCBI SRA database and are publicly available. Accession numbers are listed in the key resources table.

## Acknowledgments

We deeply appreciate the time and effort of all study participants and everyone who contributed to this work. The authors gratefully acknowledge the financial support from the 10.13039/501100001809National Natural Science Foundation of China grants 32400153 (to Y.P.), 32070133, and 32470111 (to L.F.); Scientific Research Project of 10.13039/501100010882Tianjin Municipal Education Commission grant no. 2024ZXZD016 (to M.Z.); 10.13039/501100004791Shenzhen Science and Technology Program, JCYJ20210324135007019 (to L.F.); 10.13039/501100006606Tianjin Natural Science Foundation Project, 25JCZDJC01050 (to M.Z.); Key Labortory Major Project of Tianjin Grant, 25ZXZSSS00710 (to Y.P.); and the Fundamental Research Funds for the Central Universities, 63261043 (to Y.P.).

## Author contributions

Conceptualization, M.Z. and Y.P.; data curation and writing – original draft preparation, Q.W. and X.G.; funding acquisition, L.F., M.Z., and Y.P.; investigation, Q.W., X.G., J.Q., J.M., R.L., Xiaoya Li, Xueping Li, J.C., and L.F.; writing – review and editing, M.Z. and Y.P.; all authors have read and agreed to the published version of the manuscript. Q.W. and X.G. contributed equally to this work.

## Declaration of interests

The authors declare that they have no conflicts of interest.

## STAR★Methods

### Key resources table

**Table:** 

REAGENT or RESOURCE	SOURCE	IDENTIFIER
**Antibodies**		

Rabbit anti-Dnak	Abcam	Cat#ab69617; RRID: AB_1209209
Mouse anti-FLAG M2 antibody	Sigma-Aldrich	Cat# F1804; RRID: AB_262044
Goat Anti-Rabbit IgG	Abcam	Cat# Ab6721; RRID: AB_955447
Goat Anti-Mouse IgG	Abcam	Cat# Ab205719; RRID: AB_2755049

**Bacterial and virus strains**		

CFT073	American Type Culture Collection and the National Collection of Type Cultures (ATCC)	Cat# 700928

**Chemicals, peptides, and recombinant proteins**		

Luria-Bertani (LB) broth	Sangon Biotech	Cat# A507002
RPMI 1640 medium	Gibco	Cat# 61870036
Fetal bovine serum	Gibco	Cat# 10091130
TRIzol	Invitrogen	Cat# 15596026
Chloral hydrate	Sangon Biotech	Cat# A600288
Kanamycin sulfate	Adamas	Cat# 41808D
PMSF	Invitrogen	Cat# 36978
SYBR Green PCR Master Mix	Applied Biosystems	Cat#4367659
PrimeSTAR Max DNA polymerase	TaKaRa	Cat# R045A
Premix Taq™	TaKaRa	Cat# RR902A
Protease inhibitors	Roche	Cat# 4693116001
IPTG	Sigma-Aldrich	Cat# I6758
10×Loading Buffer	TaKaRa	Cat# SD6079
2×SuperNova PCR Mix	Genstar	Cat# ZA064-101S
50×TAE Buffer	Solarbio	Cat# T1060
20×TBST Buffer	Solarbio	Cat# T1082
10×TBE Buffer	Solarbio	Cat# T1051
2×Protein Native PAGE Loading Buffer	TaKaRa	Cat# 9174
Ni Sepharose High-Performance Resin	GE Healthcare	Cat# G-17-5318
2×Protein Native PAGE Loading Buffer	TaKaRa	Cat# 9174
TEMED	Shyuanye	Cat# R21208

**Critical commercial assays**		

TIANamp Bacteria DNA Kit	TIANGEN	Cat# DP302-02
TIANpure Mini Plasmid Kit	TIANGEN	Cat# DP104-02
StarPrep DNA Gel Extraction Kit	GeneStar	Cat# D205-04
RNase-free DNase set	QIAGEN	Cat# 79254
MinElutePCR Purification Kit	QIAGEN	Cat# No.28006

**Deposited data**		

Raw and analyzed data	This paper	Sequence read archive database: SRA, PRJNA1277113; [Database]: https://doi.org/10.5281/zenodo.19679944

**Experimental models: cell lines**		

5367	American Type Culture Collection and the National Collection of Type Cultures (ATCC)	Cat# HTB-9

**Experimental models: organisms/strains**		

Mouse: BALB/c	Charles River	Cat# 211

**Oligonucleotides**		

Primers for this study, see Table S2	This paper	N/A

**Recombinant DNA**		

Plasmids for this study, see Table S1	This paper	N/A

**Software and algorithms**		

GraphPad Prism 9.0.1	GraphPad Software	https://www.graphpad.com/
ZEN	Zeiss	https://www.zeiss.com/microscopy/us/products/microscope-software.html
ImageJ 1.50	ImageJ	https://imagej.net/
DESeq2	Bioconductor	https://bioconductor.org/packages/release/bioc/html/DESeq2.html

### Experimental model and study participant details

#### Animal experiments

All the animal experiments were conducted in accordance with the standards set forth in the Guide for the Care and Use of Laboratory Animals. The experimental protocol was approved by the Animal Protection Committee of the Nankai University, Tianjin, China. The approval number for previously conducted experiments, 2023-SYDWLL-000080, expires on February 26, 2026; experiments conducted thereafter are covered by approval number 2026-SYDWLL-000566. Maximum effort was made to minimize animal suffering and limit the number of animals used. Six-week-old female BALB/c mice (Beijing Vital River Laboratory Animal Technology) were used for the infection experiments. Six-week-old female C3H mice (Beijing Vital River Laboratory Animal Technology) were used for the IBCs enumeration experiments. Mice were housed under specific pathogen-free conditions and fed a standard mouse chow diet. 6- to 8-week-old female BALB/c or C3H mice were used for experiments, in alignment with the pronounced clinical sex bias for UTIs. This design limited the assessment of sex-based differences. All infections were performed in a biosafety level two facility and following the approved protocols.

#### Cell culture conditions

Human bladder epithelial cell 5637 (ATCC HTB-9) was purchased from the American Type Culture Collection (Manassas, USA) and cultured in RPMI 1640 medium (Gibco; 61870036) containing 10% fetal bovine serum (FBS, Gibco; 10091130) at 37 °C in a 5% CO2 incubator. ATCC provides standard characterization. Mycoplasma testing (negative, tested monthly).

#### Bacterial strains and plasmids

The bacterial strains, plasmids, and primers used in this study are listed in Tables S1 and S2; uropathogenic *Escherichia coli* CFT073 (O6: K2: H1) was used as the WT strain. The gene deletion strain was generated by replacing the target gene with a resistance gene using λ-Red recombinase. The complementary strain Δ*rstAB*+p*rstAB* was generated by transforming Δ*rstAB* with a pACYC184 recombinant plasmid carrying the *rstAB* genes and its own promoter. The recombinant plasmid of *rstA* ligated to pET-28a (+) was transformed into *E. coli* BL21 (DE3) to obtain a strain expressing purified RstA protein. The pET-Duet and GFP-conjugated recombinant plasmids were transformed into WT and Δ*rstAB* to obtain strains for laser confocal microscopy observation. The recombinant fragments linking the amplified kana resistance fragment and the 3x Flag coding sequence were transformed by homologous recombination into the FimH region of the target genes of the WT, Δ*rstAB*, and Δ*rstAB*+p*rstAB* strains to obtain the FimH-Flag strains. The recombinant fragments linking the amplified kana resistance fragment and the *fimS* phase-locked-ON (left inverted repeat, LIR) coding sequences were transformed by homologous recombination into the *fimS* region of the target genes of the WT and Δ*rstAB* strains to obtain the LIR strains. Transcriptional fusions were constructed by linking the UPEC *fimS* and *fimS*-mut promoters with the lacZ reporter gene lacking a promoter, used to monitor *fimS* and *fimS*-mut expression, respectively. Promoter fragments were cloned into the EcoRI and BamHI sites of the pMLB1034 vector, yielding p*fimS* and p*fimS*-mut. These plasmids were transformed into WT and Δ*rstAB* strains, and the transcriptional activity of *fimS* and *fimS*-mut was monitored by measuring β-galactosidase activity.^70^ As a negative control, WT strains carrying pMLB1034 were used. The recombinant fragments linking the amplified kana resistance fragment and the 3 x Flag coding sequence were transformed by homologous recombination into the *rstA* or *rstB* region of the target genes of the WT strains to obtain the WT*-rstA*-Flag and WT-*rstB*-Flag strains. The strains were cultured in LB broth at 37 °C. The medium was supplemented with the appropriate antibiotics: chloramphenicol, 25 μg/mL; ampicillin, 50 μg/mL; and kanamycin, 50 μg/mL. Identical inoculum pretreatment and operational procedures were applied for WT vs. Δ*rstAB* vs. complement mutant experiments within the same group.

### Method details

#### Mouse infection assays

Bacteria in the overnight cultures were centrifuged and resuspended in PBS, and the bacterial concentration was adjusted to 3 × 10^8^ CFU/mL using a spectrophotometer. Mice were anesthetized using intraperitoneal injection of 70 mg/kg Pentobarbital Sodium (Sigma-Aldrich; 57-33-0). Each group of mice was injected with 50 μL of bacterial suspension (approximately 1 × 10^7^ CFU) via urethral catheter. Infected mice were sacrificed through cervical dislocation, and their bladders were collected at 1, 6, or 24 h p.i. For mouse colonization experiments, at 6 or 24 h p.i., bladder tissues were ground and homogenized with sterile PBS. For the mouse invasion experiments, at 1 h p.i., bladder lining samples were exposed to 100 μg/mL gentamicin sulfate for 30 min to kill extracellular bacteria in the bladder epithelium. Gradient-diluted homogenates were spread on LB solid medium containing the relevant antibiotics for CFU counting. The mouse infection assays were calculated from three independent experiments and nine mice per group. The mouse infection assay was conducted by at least two different researchers who did not know the situation and results of the study in advance and was repeated on two independent days. Data from these experimental replicates were then combined for a comprehensive statistical analysis.

#### Growth curve construction

The strains cultured overnight at 37 °C in LB medium were washed three times with PBS and diluted 1:1000 in LB medium, RPMI 1640 or M9 medium supplemented with specific components (3% mannose) without antibiotics. Then, 200 μL per well was added to 96-well plates, and six replicate wells were set up for each strain. The incubation measurement was carried out in a multifunctional microplate reader (TECAN; Spark 10M) for 24 h, and the absorbance value was determined at 37°C and a wavelength of 600 nm. The growth curve was plotted at the end of the measurement and the experiment was performed independently three times.

#### 5637 invasion assays

5637 cells (ATCC HTB-9) were purchased from American Type Culture Collection and cultured in RPMI 1640 medium (CELLMAX; JYC11875500BT) containing 10% fetal bovine serum (Gibco; 10091130) at 37 °C, 5% CO_2_, with 1% streptomycin and 1% penicillin as necessary. Subsequently, 1×10^6^ 5637 cells were inoculated in each well of a 12-well plate. Overnight cultures were washed thrice with PBS and resuspended in RPMI 1640 to infect the cells at an MOI of 100, and 3 wells were set up for each strain in parallel for labeling. Then, cells were incubated at 37 °C under 5% CO_2_ for 30 min. The cells were washed three times with PBS and RPMI 1640 medium containing 100 mg/mL gentamicin for 30 min to kill extracellular bacteria. Cells were lysed using PBS and 1% Triton X-100 and coated with appropriate gradient dilutions of resistant LB solid medium for bacterial CFU/mL counts.

#### Lactatedehydrogenase (LDH) cytotoxicity analysis

5637 cells were cultured in 96-well plates overnight. The cells were infected with the indicated strains at an MOI of 10. After 0.5 h, culture media were replaced by medium with 100 μg/mL gentamicin sulfate for an additional 0.5 h to kill extracellular bacteria, the supernatants were collected, and the amounts of LDH were measured spectrophotometrically using a CytoTox 96 Non-Radioactive Cytotoxicity Assay Kit (Promega; G1780). Cytotoxicity (%) was calculated by comparing the LDH in culture supernatants to the total cellular LDH (the amount of LDH released upon cell lysis with lysis buffer).

#### IBC enumeration

Six-week-old female C3H/HeN mice (Beijing Vital River Laboratory Animal Technology) were infected transurethrally with 3 × 10^7^ CFU/mL of the GFP-expressing WT, Δ*rstAB* or Δ*rstAB*+p*rstAB*. A total of 27 mice were randomly divided into different infection groups (9 mice per group). At 6 h p.i., the infected mice were cervically dislocated, and their bladders were removed. Bladder linings were exposed to 100 μg/mL gentamicin sulfate for 30 min to kill bacteria outside the bladder epithelium. The bladder was unfolded, fixed on a silicone bladder staple pad, fixed with 4% paraformaldehyde for 30 min at room temperature, and rinsed thrice with PBS. The bladder was permeabilized with 0.1% Triton X-100 for 20 min at room temperature and washed thrice with PBS. Then, the samples were stained with 1 mg/mL wheat germ agglutinin (WGA, Invitrogen; W32464) for 15 min under light-avoidance conditions and washed three times with PBS. Sections were blocked with an antifade mounting medium containing DAPI (Bioss; C02-04002). The number of whole bladder IBCs per mouse was counted by observation using a confocal microscope from top to bottom and from left to right (Zeiss confocal microscope Carl Zeiss; LSM800).

#### RNA extraction and quantitative RT-PCR (real-time qPCR)

Overnight cultures of WT and Δ*rstAB* were washed three times with PBS and resuspended in RPMI-1640 to infect the cells 1 h at an MOI of 100. Then, extracellular bacteria were killed with 100 μg/mL gentamicin for 30 min. Samples were collected for total RNA extraction.

Overnight cultures of WT and Δ*rstAB* were washed three times with PBS and resuspended in PBS. Each mouse was colonized through a urethral catheter containing approximately 1 × 10^7^ CFU of bacteria. The bladder was removed at 1 h p.i., and the extracellular bacteria of the bladder epithelium were killed with 100 μg/mL gentamicin for 30 min. The samples were collected for total RNA extraction.

Total RNA was isolated from the bacteria using the TRIzol (Invitrogen; 15596018) extraction method. DNA contamination was removed using an RNA-free digestion kit (Qiagen; 79254). The quality of the RNA was evaluated using 1% agarose gel electrophoresis, and the Nanodrop 2000 was used to check the concentration and purity of extracted total RNA and to ensure that all RNA samples had A260/A280 ratios between 1.9 and 2.1, that the amount of total RNA was ≥0.5 μg, and had a concentration ≥50 ng/μL. Total RNA was extracted from the intracellular bacteria using a MICROBEnrich kit (Thermo Fisher Scientific; AM1901) to remove rRNA. The extracted RNA was reverse-transcribed into cDNA using the StarScript III RT Master Mix kit (Genstar; A233). cDNA was used as a template and mixed with forward and reverse primers (Table S1). The results were determined using the Applied Biosystems 7500 Real-Time PCR System and RealStar Fast SYBR qPCR Mix (Genstar; A304-10). All data were normalized to *gyrA* expression levels, and the fold-change in expression of the target gene relative to *gyrA* was determined using the cycle threshold method (2^−ΔΔCt^) calculation. At least three biological replicates were used for each real-time qPCR assay. Differences between groups were analyzed using Student’s *t* test.

#### RNA sequencing

Total RNA was isolated as previously described. RNA library preparation and high-throughput sequencing were performed by Major Bio, Inc. (Shanghai, China). Raw sequencing reads were mapped to the UPEC CFT073 reference genome (genome version: GCF_014262945.1) using Qubit 4.0. All sequencing data were deposited in the NCBI for Biotechnology Information SRA database under the accession code PRJNA1277113. For this analysis, we used p-adjustment as the threshold for screening DEGs. The DESeq2 software, based on the negative binomial distribution, was used for statistical analysis of raw counts with default parameters (p-adjust <0.05;|log2FC| ≥ 1).

#### Hemagglutination assay

HA is an immunological method for titrating viruses based on its ability to bind to erythrocyte surface receptors and cause viral agglutination. Strains cultured overnight in LB medium at 37 °C were washed three times with PBS and resuspended in PBS. A 96-well plate was prepared by adding 25 μL of equal gradient dilutions of PBS bacteria to each well, followed by the addition of 25 μL of a 1% guinea pig erythrocyte suspension. The plates were fully mixed and incubated at 4 °C for 18 h. Hemagglutination titers were the maximum dilutions that caused significant agglutination of guinea pig erythrocytes.

#### Western blot analysis

After sonication, the samples were boiled with SDS loading buffer (Takara, 9173). Proteins were separated using 12% sodium dodecyl sulfate-polyacrylamide gel electrophoresis and transferred to polyvinylidene fluoride (PVDF) membranes. The membrane was blocked with a 5% skim milk powder blocking solution at room temperature for 1 h. Then, the membrane was incubated with primary antibodies of anti-DnaK (Abcam; Ab69617) and anti-Flag (Abcam; F1804) overnight at 4 °C. The range of protein marker (Genstar; ZM228-101) is 10-250 kDa. After the membrane was incubated with the corresponding secondary antibodies for 1 h at room temperature, the proteins were imaged with Enhanced Chemiluminescence (ECL) western blotting detection reagent (ChemiDoc MP Bio-Rad). Finally, the protein band density was calculated using ImageJ software.

#### Protein expression and purification

A recombinant pET-28a (+) plasmid with *rstA* DNA fragments was constructed and transformed into *E. coli* BL21 (DE3). The culture was induced with 0.1 mM IPTG (Sigma-Aldrich; I6758) at 16 °C for 16 h. The cultures were centrifuged and resuspended in PBS with the addition of 1 mM PMSF (Invitrogen, 36978), 5 mg/mL DNase (Invitrogen, 18047019), 10 mg/mL RNase (Invitrogen, AM2295), and 1 mg/mL RNase (Invitrogen, AM2295) and 1 mg/mL lysozyme, and the supernatant was collected using centrifugation at 12,000 g for 20 min after sonication. Affinity purification of the tagged proteins was performed using a Ni Sepharose column (GE Healthcare; AC17629), followed by anion exchange using a Mono Q column (Sigma-Aldrich; 54807) in stocking buffer (50 mM Tris-HCl, pH 8.0, 10% [v/v] glycerol, and 100 mM NaCl). Finally, protein concentration and purity were examined using a BCA Protein Assay Kit (Thermo Fisher Scientific; 23225) and stained with Coomassie Blue Staining Solution (Beyotime; P0017B).

#### Electrophoretic mobility shift assay (EMSA)

The 6 × His-tagged RstA protein was expressed and purified in *E. coli* BL21 (DE3), and the *fimS* (284 bp) and *fimS-mut* (284 bp) promoter DNA probes were amplified using CFT073 chromosomal DNA as a template. A pair of primers was designed for amplification of the *kana* DNA fragment as a negative control (primers listed in Table S1). The amplification products were purified using a GenStar PCR product purification kit (Sangon; B518141-0050). The purified fragment (40 ng) was incubated with RstA protein in a 20 μL system of binding buffer at 25 °C for 30 min. The concentration of proteins used were 0 μM, 0.25 μM, 0.5 μM, 1 μM, 1.5 μm, and 2 μM. The binding buffer was 10 mM Tris (pH 7.5), 1 mM dithiothreitol, 50 mM KCl, 10 mM MgCl_2_, 10 mM acetyl phosphate, 1 μg/mL bovine serum albumin, 1 mM EDTA, and 5% glycerol. The reaction mixture was electrophoresed on an 8% native polyacrylamide gel at 80 V for 2 h. The electrophoresis buffer was 0.5× TBE. Finally, the gels were stained with 0.1% Gel Red (Beyotime; D0139) for 5 min and visualized using a Tanon Gel Imaging System.

#### β-Galactosidase assays

Transcriptional expression analysis using several fusion constructs (promoters from *fimS*, and *fimS*-mut fused to the β-galactosidase reporter gene) were cultured statically for 12 h at 37°C. The cultures were diluted 1:50 in fresh LB and cultivated at 37°C to an OD600 of 0.65–0.70. The cultures were then diluted 1:5 in Z buffer (0.06 M Na2HPO4, 0.04 M Na2HPO4, 0.01 M KCl, 0.001 M MgSO4, and 0.05 M β-mercaptoethanol) and the β-galactosidase activity was assayed using ONPG (Yeasen; 60343ES03) as a substrate. Samples were incubated at 28°C, and the reaction was terminated by adding Na_2_CO_3_ (300 mM). Absorbance at 420 nm was measured spectrophotometrically.^70^ The β-galactosidase assay was repeated at least three times, with four parallel samples per replicate.

#### Assay for *fimS* orientation

Stentebjerg-Olesen et al. described a method for determining the orientation of *fimS* phase inversion using restriction enzyme digestion of PCR-amplified *fimS* fragments.^57^ Genomic DNA was extracted from WT and Δ*rstAB* after 24 h of static cultivation in LB medium. The *fimS* invertible element (601 bp) was amplified using PCR with the following primers (Table S1) F: 5ʹ-CAGTAATGCTGCTCGTTTTGCCG-3ʹ and R: 5ʹ-CAGAGCCGACAGAACAACG-3ʹ. The range of DNA marker (Takara; 3427A) is 100-2000bp. The restriction enzyme SnaBI cleaves an asymmetric site within *fimS*. When *fimS* is in the ON orientation, digestion fragments of 403 bp and 198 bp are produced, whereas cleavage of *fimS* in the OFF orientation produces 440-bp and 161-bp fragments. The size and band intensity of the digested fragments were evaluated using 1% agarose gel electrophoresis and visualized using ultraviolet transillumination.

### Quantification and statistical analysis

All experiments were repeated three times. Quantitative data are shown as the mean ± standard deviation (SD) (*n* = 3). *p* values between the two experimental groups were calculated using the unpaired Student’s *t* test. For 1 h, 6 h, and 24 h p.i. mouse infection experiments, data were obtained from three independent experiments and presented as the mean ± SD, and the Mann-Whitney *U* test was used. *p* ≤ 0.05 was considered statistically significant. Data were analyzed using GraphPad Prism (GraphPad Software, La Jolla, CA, USA) version 7.00; La Jolla, CA, USA). *Fisher*’s exact test and false discovery rate (FDR) multiple test corrections were used to identify significant GO and KEGG categories.

## Supplemental information

Document S1. Figures S1–S5 and Tables S1 and S3

Table S2. Primers used in this study

Data S1. Original ethical approval document
