# Supplementary material for: RstAB activates type 1 fimbriae to promote uropathogenic Escherichia coli bladder invasion
Source: iScience. 2026 Jun 11;29(7):116333. doi: 10.1016/j.isci.2026.116333 (PMC13276309; doi:10.1016/j.isci.2026.116333)
Supplement: Document S1. Figures S1–S5 and Tables S1 and S3 [file mmc1.pdf]

iScience, Volume *29*

## Supplemental information

# RstAB activates type 1 fimbriae to promote uropathogenic *Escherichia coli* bladder invasion

**Qian Wang, Xinyu Gao, Jiamin Qian, Jianwei Mu, Ruiying Liu, Xiaoya Li, Xueping Li, Chen Jin, Lu Feng, Mingqing Zhang, and Yu Pang**

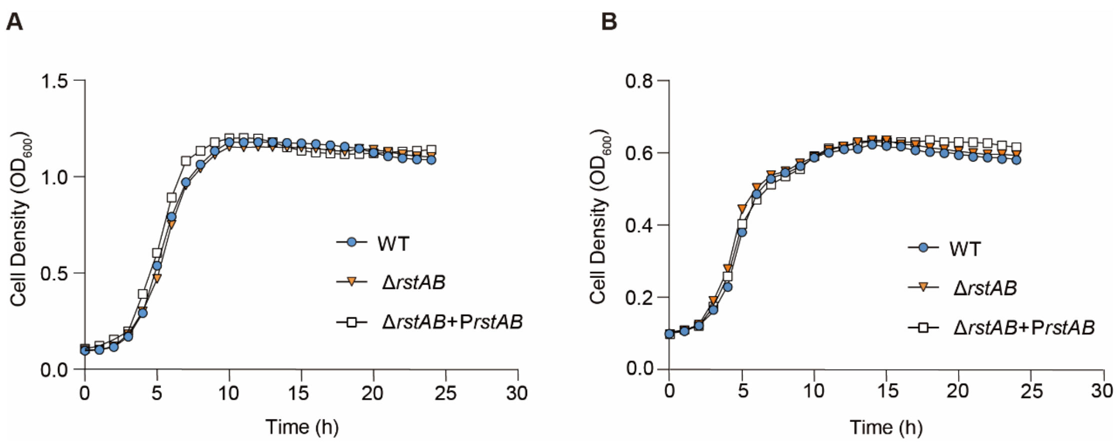


**Fig. S1 RstAB contributes to UPEC colonization in mouse bladder independently of a growth defect. Related to Fig. 1**

(A, B) Growth curves of WT, WT, Δ*rstAB*, or Δ*rstAB*+P*rstAB* in LB medium (A) and RPMI 1640 medium (B). The absorbance of bacterial suspensions at 600 nm (OD600) was measured regularly using a microplate reader over a 24 h period.

Data are presented as mean ± SD, n = 3 independent experiments.

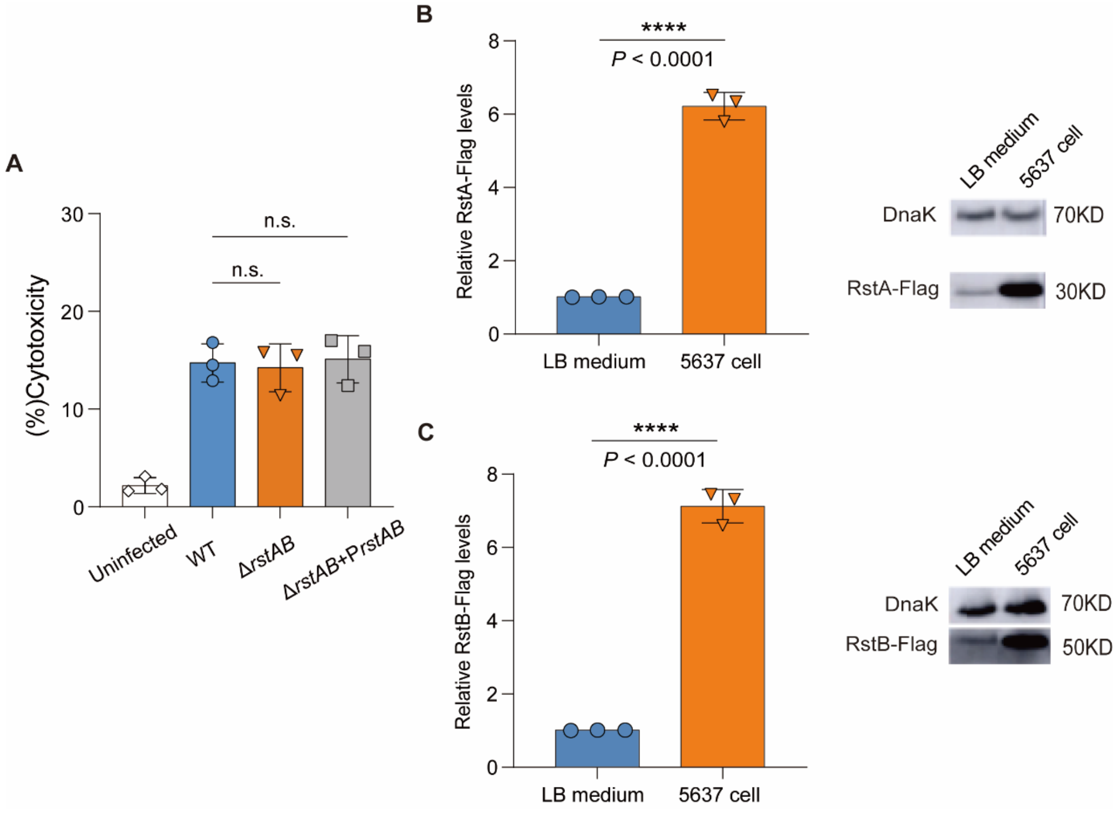


**Fig. S2 RstA and RstB expression is upregulated during host invasion. Related to Fig. 1**

(A) 5637 cells were infected with WT, Δ*rstAB*, or Δ*rstAB*+P*rstAB* for 1 h p.i. Cell death

was determined by LDH release.

(B, C) Western blot analysis shows the expression levels of rstA-Flag (A)and *rstB*-Flag (B) proteins in WT strains 1 h p.i. of 5637 cells.

DnaK was used as the loading control. Representative image from three independent experiments (B and C). *P* values were determined using two-tailed Student's *t*-test (A, B and C). ****$P \leq 0.0001$; n.s., No significant difference.

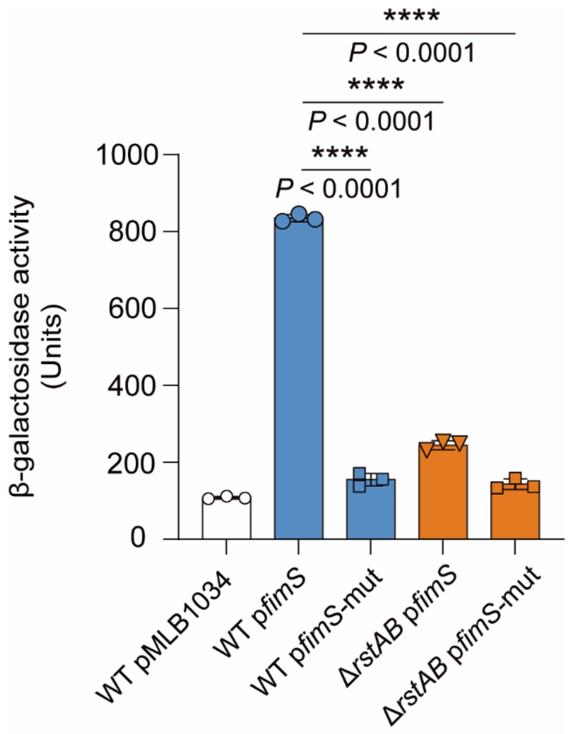


**Fig. S3 RstA binding site is on *fimS*. Related to Fig. 4**

Fusion constructs consisting of *fimS* and *fimS*-mut promoters that had been fused to the promoter-less β-galactosidase reporter gene were employed to quantitatively determine the activity of *fimS* and *fimS*-mut promoters.

Data were obtained from three independent experiments and presented as mean ± SD. *P* values were determined using two-tailed Student's *t*-test. ****$P \leq 0.0001$.

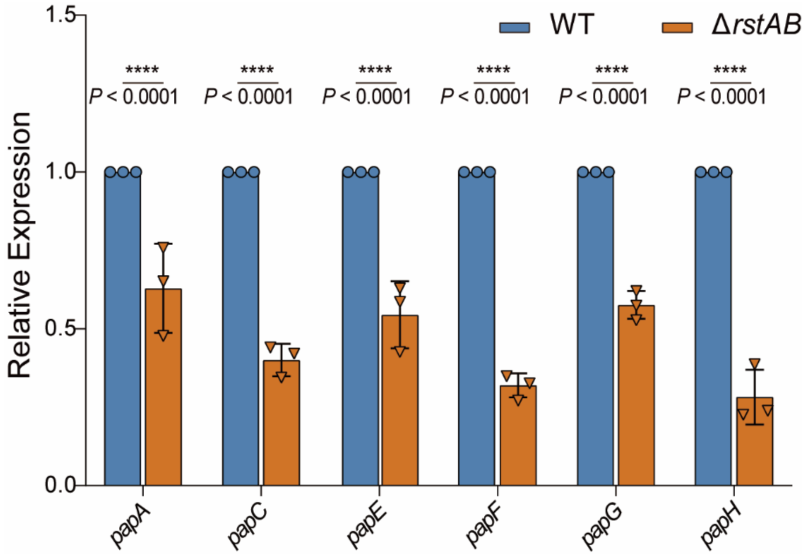


**Fig. S4 RstAB positively regulates *pap* gene expression *in vitro*. Related to Fig. 5**

qRT-PCR analyses of the mRNA levels of *papA*, *papC*, *papE*, *papF*, *papG* and *papH* in WT or Δ*rstAB* statically cultured in LB medium for 12 h.

Data were obtained from three independent experiments and presented as mean ± SD. Significance was determined using two-tailed unpaired Student's *t*-test. Significance was indicated by *P* value. **** $P \leq 0.0001$; n.s. represents no significant difference.

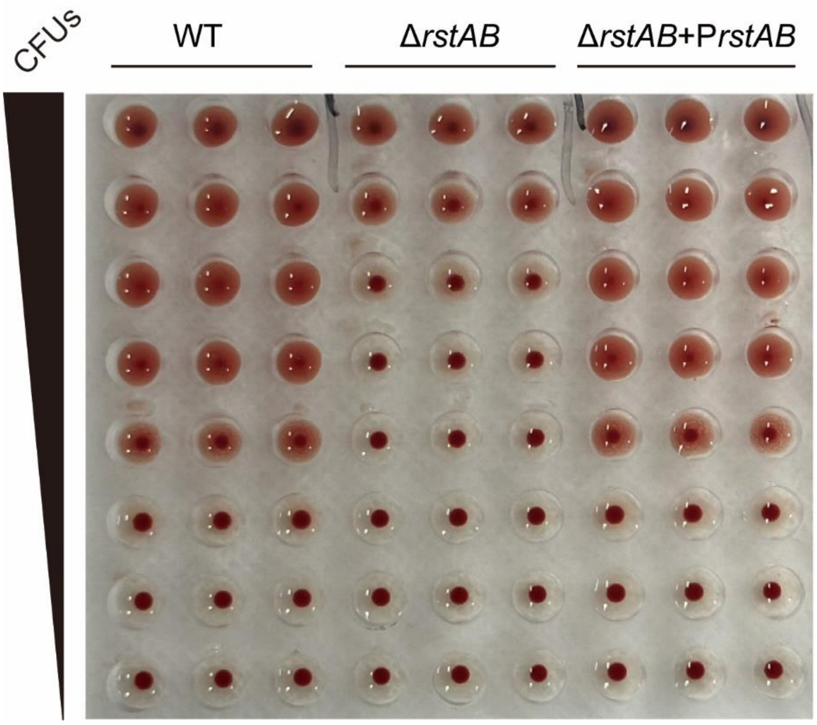


**Fig. S5 RstAB promotes the surface expression of type 1 fimbria. Related to Fig. 3**

**Table S1 Plasmids and strains used in this study. (related to STAR Methods)**

| **Plasmids** | | |
|---|---|---|
| pKD3 | Carrying the chloramphenicol acetyltransferase gene | Lab collection |
| pSim5 | Providing λ Red recombinase system | Lab collection |
| pACYC184 | Carrying bacteria expression vector with low copy number | Lab collection |
| pACYC184-*rstA* | pACYC184 carrying *rstA* gene and its promoter region | This study |
| pETDuet | Carrying the Ampicillin gene | Lab collection |
| pETDuet-GFP | pETDuet carrying *gfp* gene | This study |
| pETDuet-*rstA*-GFP | pETDuet carrying *rstA*-*gfp* gene | This study |
| pET-28a (+) | Providing the DNA sequence of 6×His tags | Lab collection |
| pET-28a (+)-*rstA* | pET-28a (+) carrying *rstA* gene and its promoter region | This study |
| pMLB1034 | Providing the DNA sequence of LacZ | Lab collection |
| pMLB1034-*fimS* | pMLB1034 carrying *fimS* gene | This study |
| pMLB1034-*fimS*-mut | pMLB1034 carrying *fimS*-mut gene | This study |
| **Strains** | **Genotype or description** | **Source** |
| BL21 | Expression strain | Lab collection |
| DH5α | *E. coli* DH5α/λpir strain | Lab collection |
| WT | Uropathogenic *Escherichia coli* strain CFT073 (O6: K2: H1) | Lab collection |
| Δ*rstAB* | *rstAB* deletion mutant in CFT073 | This study |
| Δ*rstAB*+p*rstAB* | *ΔrstAB* containing pACYC184-*rstA* | This study |
| Δ*fimA-H* | *fimACDFGHI* deletion mutant in CFT073 | This study |
| Δ*fimA-H*Δ*rstAB* | *fimACDFGHI and rstAB* deletion mutant in CFT073 | This study |
| 6x His tag *rstA* | BL21 containing pET-28a (+)-*rstA* | This study |
| WT-GFP | CFT073 containing pETDuet expressing *gfp* gene | This study |
| Δ*rstAB*-GFP | Δ*rstAB* containing pETDuet expressing *gfp* gene | This study |
| Δ*rstAB*+p*rstAB*-GFP | Δ*rstAB*+p*rstAB* containing pETDuet expressing *gfp* gene | This study |
| WT+*rstA*-GFP | CFT073 containing pETDuet expressing *rstA* promoter-*gfp* gene | This study |
| WT-LIR | CFT073 *fimS* left inverted repeat, | This study |
| Δ*rstAB*-LIR | Δ*rstAB fimS* left inverted repeat, | This study |
| WT-*fimH*-Flag | CFT073 containing *fimH*-3 x Flag | This study |
| Δ*rstAB fimH*-Flag | Δ*rstAB* containing *fimH*-3 x Flag | This study |
| Δ*rstAB*+p*rstAB*-*fimH*-Flag | Δ*rstAB*+p*rstAB* containing *fimH*-3 x Flag | This study |
| Δ*rstA*-p*fimS* | Δ*rstA* containing pMLB1034-*fimS* | This study |
| Δ*rstA*-p*fimS*-mut | Δ*rstA* containing pMLB1034-*fimS*-mut | This study |
| WT- p*fimS* | WT containing pMLB1034-*fimS* | This study |
| WT-p*fimS*-mut | WT containing pMLB1034-*fimS*-mut | This study |
| *rstA-Flag* | WT containing *rstA*-Flag | This study |
| *rstB-Flag* | WT containing *rstB*-Flag | This study |
| Δ*papG* | *papG* deletion mutant in CFT073 | This study |

**Table S3 The expression of Sulfur metabolism genes were down-regulated in Δ*rstAB* compared with that in the WT. (related to STAR Methods)**

| seq_id | gene_name | rstAB count | WT count | log2fc (rstAB/wt) | *p*value | Regulate (rstAB/wt) |
|---|---|---|---|---|---|---|
| HGG68_RS15305 | *cysC* | 6 | 30 | 0.23673575 | 0.000185224 | down |
| HGG68_RS15315 | *cysD* | 20 | 53 | 0.446671227 | 0.001124062 | down |
| HGG68_RS15335 | *cysI* | 51 | 248 | 0.243417808 | 2.55214E-26 | down |
| HGG68_RS15340 | *cysJ* | 161 | 414 | 0.460319514 | 1.0417E-18 | down |
| HGG68_RS13590 | *cysA* | 81 | 200 | 0.479389894 | 4.20797E-09 | down |
| HGG68_RS13595 | *cysW* | 31 | 78 | 0.470436426 | 0.000179806 | down |
| HGG68_RS17805 | *cyuP* | 484 | 1354 | 0.423117072 | 2.52803E-67 | down |
