# Supplementary material for: RstAB activates type 1 fimbriae to promote uropathogenic Escherichia coli bladder invasion
Source: iScience. 2026 Jun 11;29(7):116333. doi: 10.1016/j.isci.2026.116333 (PMC13276309; doi:10.1016/j.isci.2026.116333)
Supplement: Data S1. Original ethical approval document [file mmc3.zip › 20260430 original ethical approval document -qian wang.pdf]

实验动物使用伦理审查申请表

审批号：2026-SYDWLL-000566

**一、项目申请基本情况**			
项目名称	尿路致病性大肠杆菌双组份系统 RstA/RstB 的功能研究		
项目申请人	王倩	所属二级单位	南开大学第一附属医院
职称	研究实习员	联系电话	18716023115
申请审查类型	☑申请项目 □批准后项目 □延续项目 □委托项目		
**二、所需实验动物**			
种类	C3H/HeN，BALB/c 小鼠	年龄	6-8 周
体重/大小	18g	性别	不限
来源	北京维通利华实验动物技术有限公司		
饲养场所	南开大学泰达生物技术研究院	实验场所	南开大学泰达生物技术研究院
数量	__150__ + __200__ + __150__ = __500__<br>（第一年） （第二年） （第三年） （总计）		

三、动物运送（是否需要运送，如需请说明运送路线及使用工具等）

维通利华公司负责实验动物的运送。运输采用实验动物专用运输车，由北京直接运送至南开大学泰达生物技术院。

四、研究目的（简单描述研究目的及该研究对人类、动物、科学研究的贡献）

尿路感染（urinary tract infection, UTI）是一种常见的女性多发性疾病，大约每年患病人数达 1.5 亿。革兰氏阴性和阳性细菌以及一些真菌均可引起 UTI，其中 75-95%的 UTI 是由尿路致病性大肠杆菌（uropathogenic *Escherichia coli*, UPEC）引起的。UPEC 致病过程中的决定因素是其能够在侵入膀胱上皮细胞后在细胞质中形成生物膜样细胞内的细菌群落（intracellular bacterial communities, IBCs），使其能够在小鼠膀胱组织内成功定植进而引起 UTI。作为 UTI 建立的第一步成功入侵 BECs 是 UPEC 致病的关键步骤，为了进一步了解 UPEC 入侵宿主的调控机制以及其 TCS 对不同环境信号的反应。

本研究旨在寻找 UTI 致病过程中关键步骤的相关功能基因及其表达调控机制。研究结果将有助于我们深入了解 UPEC 在致病过程的关键步骤的分子机理，加深我们对其致病机制的认识；将有助于我们发现新的药物靶点，研发出新型安全有效的针对 UPEC 引发的 UTI 的治疗方法。

五、使用实验动物的原理（解释使用动物的原理；阐述选择动物属种和数量的依据）

UPEC 在国际上的主要研究模型是小鼠 UTI 模型，用于解析与 UTI 相关的毒力因子和调控网络。实验动物多选择健康的 6–8 周的 C3H/HeN 或 BALB/c 雌性小鼠，对小鼠的膀胱组织定植以及 IBCs 形成的数量、大小和形态能够反映不同菌株对小鼠的致病及 IBCs 形成能力的差异。

一般情况下，突变株、野生株和回补菌株都需要进行小鼠膀胱定植以及 IBCs 形成能力实验。小鼠膀胱定植实验每种菌在需要至少 8-10 只小鼠，20 种菌的定植实验，因此预计需要 200 只小鼠。小鼠膀胱 IBCs 形成实验每种菌需要至少 8-10 只小鼠，我们需要观 20 种菌的 IBCs 形成实验，因此预计需要 300 只小鼠。总计需要使用小鼠数量为 500 只。

六、描述动物实验的设计和操作程序

实验动物为 6-8 周的雌性 C3H/HeN 或 BALB/c 小鼠，在 25℃恒温恒湿条件下饲养，喂食标准鼠粮和无菌水。稳定期的细菌，调 OD600 = 0.6 后收菌。戊巴比妥钠经由腹腔注射麻醉小鼠。50 μL 的含 2×10^8 CFU 细菌的 PBS 缓冲液经小鼠尿道注入。感染 24 h 后采用颈椎脱臼法处死小鼠，取出膀胱。用 100 μg/mL 庆大霉素的 PBS 缓冲液处理膀胱腔表面的细菌。膀胱匀浆，稀释涂布，统计膀胱中的细菌数目。

七、手术操作程序（如需手术，请填写本目录）

具体操作步骤	（1）用腹腔注射的方式给小鼠进行麻醉；<br>（2）经尿路给小鼠注入一定量的细菌；<br>（3）作用相应时间点后，用断颈法处死被感染的小鼠；<br>（3）分离小鼠的膀胱；<br>（4）研磨分离的器官；<br>（5）逐级稀释，涂平板，计算小鼠膀胱组织中的菌数。
手术操作人及培训情况	王倩，已参加过动物实验的培训
手术执行的地点	南开大学泰达生物技术研究院
实验后的护理	动物在实验前已处死
是否在同一动物上进行多个手术	☑ 否<br>☐ 是，具体说明：

八、导致疼痛的分类

☑A 轻微，或一过性，或无疼痛

☐ B 有疼痛，但能够解除

☐ C 不能缓解的疼痛

九、麻醉镇痛（对第八项目中的 B 或 C 类动物，应说明使用麻醉或镇痛的方法，包括麻醉剂的名称、剂量、使用方式和时间）

麻醉药品配置：取 0.075 g 戊巴比妥钠粉末溶于 10 mL PBS 缓冲液中混匀。使用 0.75%的戊巴比妥钠腹腔注射麻醉小鼠，根据体重以 75 μL/ 10g 的计量使用麻醉剂，作用时间持续 2-3 h。

十、实验终结后，如何终止动物的生命和如何处理动物尸体

实验小鼠采用颈椎脱臼法处死。小鼠尸体先置于灭菌袋内用高压蒸汽灭菌处理半小时，再放于专用塑料袋中打结密封，集中存放在-20℃动物尸体贮存冰柜中，由医疗废弃物处理单位统一处理。

十一、有害（毒）物质的使用（应得到安全委员会的批准）

	否	是	使用物品的名称
1 放射性同位素	_否_	____	
2 生物物品	__	_是_	尿路致病性大肠杆菌（UPEC）
3 有毒的化学（药）品	_否_	____	
4 重组 DNA	_否_	____	

生物安全水平：

二级生物安全

具体描述安全操作和处理被污染的动物和有关污染物：

按照《病原微生物实验室生物安全管理条例》及相关规章、规范性文件和技术标准、规范的规定及病原微生物实验室管理的要求，在二级生物安全防护级别实验室中，按照标准操作规程开展实验活动。

**项目申请人承诺：**<br><br>本人申请研究的项目符合有关实验动物使用伦理的相关规定。<br><br>项目申请人（签字）：王倩<br><br>2026 年 2 月 24 日
**伦理委员会审核意见：**<br><br>经审查，该研究项目符合有关实验动物使用伦理的相关规定，同意开展研究。<br><br>实验动物福利伦理审查委员会负责人（签章）：[illegible]<br><br>公章<br><br>2026 年 3 月 10 日
